# Large-scale sequencing based on full-length-enriched cDNA libraries in pigs: contribution to annotation of the pig genome draft sequence

**DOI:** 10.1186/1471-2164-13-581

**Published:** 2012-11-15

**Authors:** Hirohide Uenishi, Takeya Morozumi, Daisuke Toki, Tomoko Eguchi-Ogawa, Lauretta A Rund, Lawrence B Schook

**Affiliations:** 1 Agrogenomics Research Center, National Institute of Agrobiological Sciences, 2 Ikenodai, Tsukuba, Ibaraki, 305-8602, Japan; 2 Division of Animal Sciences, National Institute of Agrobiological Sciences, 2 Ikenodai, Tsukuba, Ibaraki, 305-8602, Japan; 3 Animal Genome Research Program, 2 Ikenodai, Tsukuba, Ibaraki, 305-8602, Japan; 4 Animal Research Division, Japan Institute of Association for Techno-innovation in Agriculture, Forestry and Fisheries, 446-1 Ippaizuka, Kamiyokoba, Tsukuba, Ibaraki, 305-0854, Japan; 5 Institute for Genomic Biology, University of Illinois at Urbana-Champaign, 1206 West Gregory Drive, Urbana, IL, 61801, USA

**Keywords:** *Sus scrofa*, Full-length cDNA, Sequencing, Genome annotation

## Abstract

**Background:**

Along with the draft sequencing of the pig genome, which has been completed by an international consortium, collection of the nucleotide sequences of genes expressed in various tissues and determination of entire cDNA sequences are necessary for investigations of gene function. The sequences of expressed genes are also useful for genome annotation, which is important for isolating the genes responsible for particular traits.

**Results:**

We performed a large-scale expressed sequence tag (EST) analysis in pigs by using 32 full-length-enriched cDNA libraries derived from 28 kinds of tissues and cells, including seven tissues (brain, cerebellum, colon, hypothalamus, inguinal lymph node, ovary, and spleen) derived from pigs that were cloned from a sow subjected to genome sequencing. We obtained more than 330,000 EST reads from the 5′-ends of the cDNA clones. Comparison with human and bovine gene catalogs revealed that the ESTs corresponded to at least 15,000 genes. cDNA clones representing contigs and singlets generated by assembly of the EST reads were subjected to full-length determination of inserts. We have finished sequencing 31,079 cDNA clones corresponding to more than 12,000 genes. Mapping of the sequences of these cDNA clones on the draft sequence of the pig genome has indicated that the clones are derived from about 15,000 independent loci on the pig genome.

**Conclusions:**

ESTs and cDNA sequences derived from full-length-enriched libraries are valuable for annotation of the draft sequence of the pig genome. This information will also contribute to the exploration of promoter sequences on the genome and to molecular biology-based analyses in pigs.

## Background

The pig is the world's most frequently consumed meat animal, and its genetic improvement, particularly in terms of productivity and meat quality, is of interest to livestock science [1]. To date, intensive genetic improvement of livestock animals has been conducted by using classical selection and mating, but genomic information is required for further improvement. Improvements in aspects of the rearing management of pigs, such as feeding and hygiene control, have to be based on knowledge obtained from physiological studies. Moreover, the pig is unique among livestock in that it is very useful in biomedical research because of the structural and size similarities of its organs (particularly cardiovascular and dermal) to those of humans [2-4]. Improvement in the breeding and rearing of pigs with the help of molecular genetics and physiology, as well as the use of pigs as biomedical model animals, requires fundamental information on pig molecular biology, particularly in terms of the genome and genes.

Recently, the International Swine Genome Sequencing Consortium (SGSC) completed its draft sequencing of the pig genome; these sequences will form the basis of further investigations of pig molecular biology [5-7]. Sequencing of the pig genome will accelerate the development of genetic markers to improve breeds and populations and give basic information on the genes encoded on the genome [8]. However, genes cannot be precisely localized on the genome solely from information on the genome sequence. Determination of precise sequences, structures, and locations requires information on the sequences of expressed genes *per se*[9]. The locations and structures of genes on the pig genome are now being explored by using automated systems or by manual inspection by annotators using the Otterlace system of the Wellcome Trust Sanger Institute to add information to databases such as Vertebrate Genome Annotation (VEGA) [10-12]. The sequences of expressed genes are also useful for genome annotation, which is important for isolating the genes responsible for particular traits.

Expressed sequence tag (EST) analyses have been conducted by many research groups in pigs and other organisms. More than 1,600,000 pig ESTs have been accumulated and registered in the public nucleotide databases, and several attempts at transcriptome analysis using next-generation DNA sequencing (NGS) have been made [13-15]. Most of the cDNA libraries constructed by using traditional methods do not cover the transcription start sites, because the limitations of cloning techniques can cause incomplete synthesis of full-length cDNA. On the other hand, the sequences of transcripts obtained by using NGS alone are reconstructed by the compilation of short reads and do not directly reflect the actual structure of the mRNA; this may be problematic in considering the alternative splicing products that are actually expressed in the tissues [16]. As far as possible, it is therefore important to clone full-length mRNA transcripts in order to collect gene expression data and use these data in further analyses of expressed genes and of genome annotation in pigs [17-19]. cDNA clones carrying full-length transcripts have additional benefits—they can be used for protein production *in vitro* and for exploring promoter sequences on the genome.

So far we have conducted EST analysis and sequencing of entire mRNA transcripts in pigs by using full-length-enriched cDNA libraries. Here, we outline the data we have collected and the advantages of their use, particularly in genome annotation of the draft sequence of the pig genome.

## Results and discussion

### Pig ESTs based on full-length-enriched cDNA libraries

**Figure 1:**
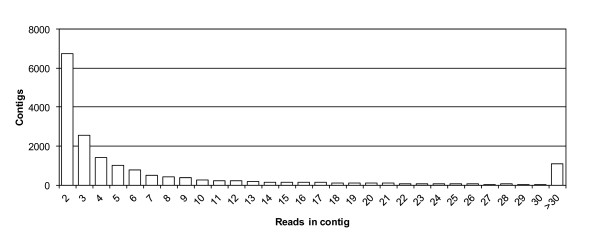
**Distribution of EST reads in contigs.** Contigs are ordered by the numbers of expressed sequence tags (ESTs) they contained. There were 6740 contigs carrying two ESTs and 1084 contigs carrying more than 30 ESTs. There were 15,432 contigs consisting of fewer than 20 ESTs.

We have constructed 32 cDNA libraries for 28 different tissues and cell populations by using cloning of cap-structured mRNA [20,21] or the SMART method [22], and we have accumulated 330,707 ESTs from 5′-ends (Table 1) including previously reported 162,631 ESTs in our pig expressed gene database [18,19]. The ESTs thus obtained were assembled into 17,183 contigs consisting of 209,779 ESTs, with 120,928 singlets remaining. The contig containing the largest number of ESTs carried 4111 ESTs with marked similarity to tubulin α1 genes. The genes encoding α-tubulin are extremely similar to each other; therefore, the ESTs encoding α-tubulins were assembled into the same contigs. On the other hand, about 90% of the contigs contained fewer than 20 ESTs (Figure 1), showing that the majority of the ESTs were based on virtually unbiased mRNA sequences. High-quality EST reads have been registered in the public nucleotide database [DDBJ:BP137499–BP173623, BP433030–BP464980, BW954997–BW985219, CJ000001–CJ039835, DB781565–DB806061, FS639971–FS656010, FS656544–FS722296, and HX201766–HX247044].

**Table 1:** Pig cDNA libraries, ESTs, and completely sequenced cDNA clones

**Library name**	**Method of library construction**	**Tissue/cell**	**Origin**^**a**^	**Vector**	**ESTs**			**Sequenced cDNA**			
					**In contigs**	**In singlets**	**Total**	**Clones**	**Mapped on Sscrofa10.2**	**Loci**	
ADR01	Oligo-capping	Adrenal gland	LWD (LWD2)	pCMVFL3	6490	3188	9678	901	861	782	
AMP01	SMART	Alveolar macrophage	LWD (LWD7)	pDNR-LIB	5459	3704	9163	1416	1345	930	
BFLT1	Oligo-capping	Brain (frontal lobe)	Duroc (2-14C)	pME18S	8104	5678	13,782	991	953	859	
BKFL1	SMART	Backfat	Landrace (L2)	pDNR-LIB	1557	7920	9477	453	418	404	
BMWN1	Vector-capping	Bone marrow	NIBS miniature	pGCAP10	5981	2492	8473	148	133	124	
CBLT1	Vector-capping	Cerebellum	Duroc (2-14C)	pGCAP10	6082	3054	9136	0	0	0	
CLNT1	Oligo-capping	Colon	Duroc (2-14C)	pME18S	6580	6782	13,362	537	512	365	
DCI01	SMART	Immature dendritic cells	Landrace (L1)	pDNR-LIB	5953	4486	10,439	887	841	748	
HTMT1	Vector-capping	Hypothalamus	Duroc (2-14C)	pGCAP10	8063	5278	13,341	1663	1474	1264	
ILNT1	Vector-capping	Inguinal lymph node	Duroc (2-14C)	pGCAP10	6321	2658	8979	0	0	0	
ITT01	Oligo-capping	Intestine	LWD (LWD2)	pCMVFL3	7272	2475	9747	1265	1206	1037	
KDN01	Oligo-capping	Kidney	LWD (LWD8)	pME18S	5873	3235	9108	748	723	663	
LNG01	Oligo-capping	Lung	LWD (LWD3)	pCMVFL3	5186	3859	9045	1331	1250	1061	
LVR01	Oligo-capping	Liver	LWD (LWD4)	pCMVFL3	7199	1815	9014	779	741	653	
LVRM1	Oligo-capping	Liver	Meishan	pCMVFL3	13,881	5051	18,932	1844	1760	1372	
MLN01	Oligo-capping	Mesenteric lymph node	LWD (LWD2)	pCMVFL3	6443	3250	9693	1176	1099	902	
MLTL1	SMART	Longissimus muscle	Landrace (L2)	pDNR-LIB	3577	4892	8469	413	388	292	
OVR01	Oligo-capping	Ovary	LWD (LWD1)	pCMVFL3	6537	2828	9365	1416	1356	1226	
OVRM1	Oligo-capping	Ovary	Meishan	pCMVFL3	12,471	7071	19,542	3309	3149	2665	
OVRT1	Oligo-capping	Ovary	Duroc (2-14C)	pME18S	9516	4340	13,856	819	790	728	
PBL01	Oligo-capping	Peripheral blood lymphocytes	LWD (LWD5)	pCMVFL3	6652	3262	9914	957	908	732	
PCT01	Oligo-capping	Placenta	LWD (LWD9)	pME18S	2175	1115	3290	161	150	142	
PST01	Oligo-capping	Prostate	LWD (LWD10)	pME18S	6813	2329	9142	691	654	596	
PTG01	Oligo-capping	Pituitary gland	LWD (LWD4)	pCMVFL3	4281	5628	9909	864	826	790	
SKNB1	Oligo-capping	Skin	Berkshire	pME18S	4894	3363	8257	687	630	534	
SMG01	Oligo-capping	Submaxillary gland	LWD (LWD2)	pCMVFL3	6944	2680	9624	458	430	361	
SPL01	Oligo-capping	Spleen	LWD (LWD1)	pCMVFL3	6793	2811	9604	1457	1397	1207	
SPLT1	Vector-capping	Spleen	Duroc (2-14C)	pGCAP10	6037	2734	8771	0	0	0	
TCH01	Oligo-capping	Trachea	LWD (LWD3)	pCMVFL3	5151	3658	8809	1412	1345	1087	
TES01	Oligo-capping	Testis	LWD (LWD6)	pME18S	7112	2962	10,074	697	669	466	
THY01	Oligo-capping	Thymus	LWD (LWD1)	pCMVFL3	7586	3704	11,290	2158	2066	1620	
UTR01	Oligo-capping	Uterus	LWD (LWD1)	pCMVFL3	6796	2626	9422	1441	1356	1180	
Total					209,779	120,928	330,707	31,079	29,430	13,894	(2993)^b^

Thirty-two cDNA libraries constructed with pig tissues and cell populations were used to generate expressed sequence tags (ESTs). Twenty-three libraries were constructed by using the oligo-capping method [21], and five were constructed by using the vector-capping [20] method. Four libraries were constructed by using the SMART method (Clontech, Palo Alto, CA, USA) [22]. ESTs in contigs are shown, as are those that had a ≥100-bp stretch of Phred quality value ≥20 and were not involved in contigs (“In singlets”). Numbers of cDNA clones that were completely sequenced (31,079 in total) are also shown. Completely sequenced cDNA clones derived from pigs cloned from a female subjected to genome sequencing by the International Swine Genome Sequencing Consortium are classified by library.

^a^ Origins are indicated as pig breeds or lines. Different animals in the same breed are designated as described in parentheses. LWD is (Landrace × Large White) × Duroc. Duroc (2-14C) designates a Duroc individual cloned from a female pig subjected to draft genome sequencing [5-7].

^b^ Loci repeated among clones derived from different libraries were removed.

### Genes and chromosomal locations corresponding to assemblies generated from pig ESTs

**Figure 2:**
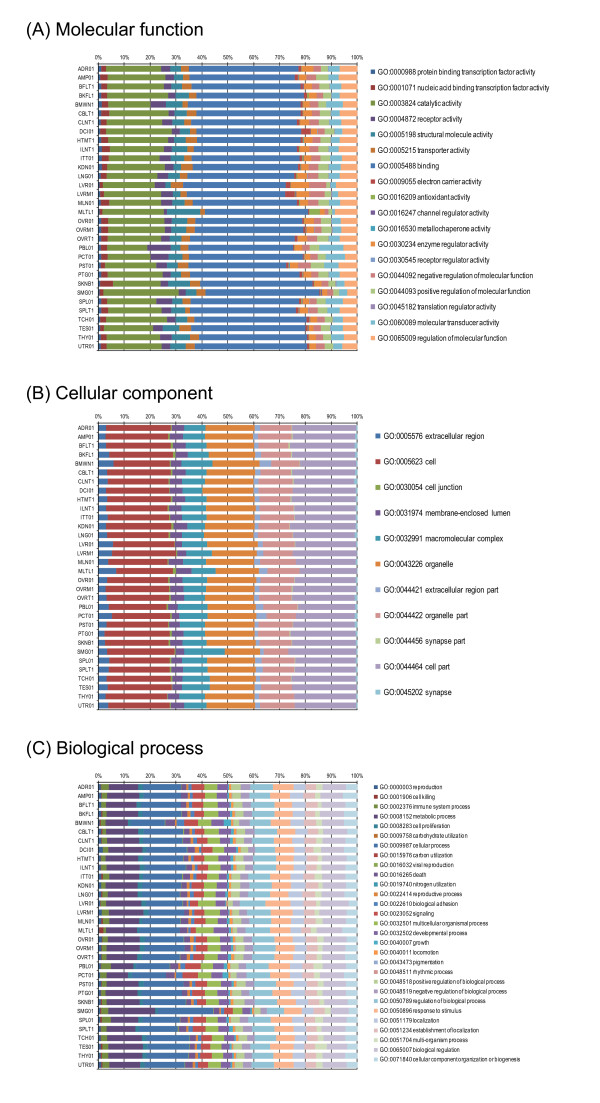
**Classification of ESTs according to Gene Ontology.** Proportions of expressed sequence tags (EST) classified according to Gene Ontology terms under the root namespaces (molecular function (**A**), cellular component (**B**), and biological process (**C**)) are indicated for each cDNA library. Classification according to Gene Ontology was conducted by using the similarity of the EST assemblies to human genes and the correspondence between genes and the Gene Ontology terms provided in NCBI Gene ( ftp://ftp.ncbi.nih.gov/gene/DATA/; [24]). ESTs classified under more than one term in a single namespace are counted redundantly under the respective terms. ESTs not classified under any terms are omitted from this figure.

**Figure 3:**
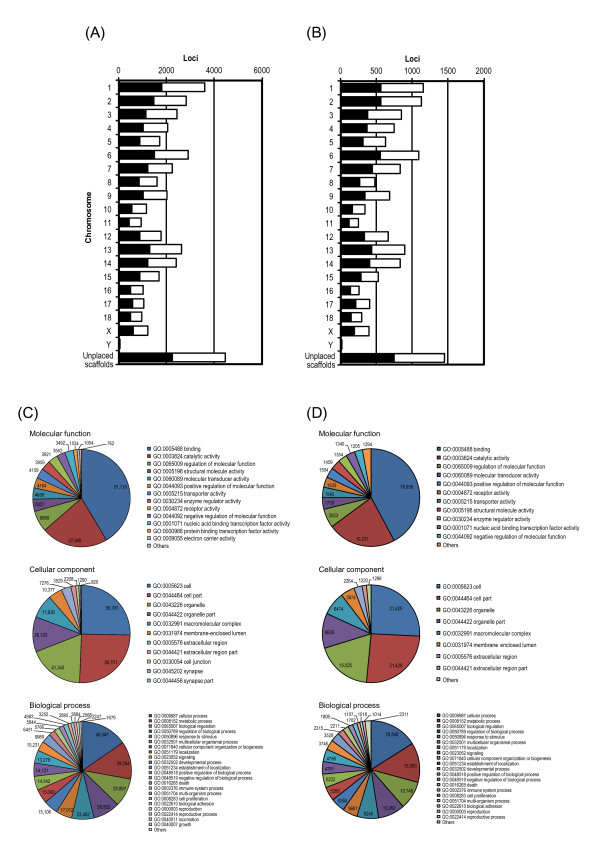
**Correspondence of EST assemblies and cDNA clones to locations on the draft sequence of the pig genome and Gene Ontology.** Shown are the numbers of loci that are located on each pig chromosome in the draft sequence of the pig genome (Sscrofa10.2) and that correspond to the EST assemblies (**A**) and pig cDNA clones (**B**) sequenced in this study. Orientations of loci are shown by closed bars (pter to qter) and open bars (qter to pter). EST assemblies (**C**) and pig cDNA clones that were completely sequenced (**D**) were classified according to the Gene Ontology terms shown under each root namespace (i.e., molecular function, cellular component, and biological process) by using the ontology file as at 31 October 2011. Classification according to Gene Ontology was conducted by using the similarity of cDNA clones to the mRNA sequences of human genes in the NCBI RefSeq (release 49) and the correspondence between genes and Gene Ontology terms provided in NCBI Gene ( ftp://ftp.ncbi.nih.gov/gene/DATA/ as at 2 November 2011; [24]). The numbers of EST assemblies classified into the three namespaces (molecular function, cellular component, and biological process) were 56,703, 59,098, and 54,610, respectively. The numbers of cDNA clones classified into the three namespaces were 21,332, 22,306, and 20,538, respectively. Assemblies and clones classified under more than one term in a single namespace are counted redundantly under the respective terms. Terms including fewer than 1000 assemblies and clones are indicated as “Others” in the aggregates.

BLAST similarity analysis [23] of the nucleotide sequences of the assemblies (contigs and singlets) against the mRNA sequences of RefSeq (release 49; 13 September 2011) at the National Center for Biotechnology Information (NCBI) [24] revealed that the cDNA sequences corresponded to 13,691 unique human genes (Table 2). Correspondence of the assemblies to the functions of human genes according to the Gene Ontology terms [25] demonstrated that the EST collection covered a broad range of porcine expressed genes (Figure 2 and 3). About three-fourths of the assemblies showing obvious similarity to protein sequences were estimated to contain start codons, indicating the high efficiency of cloning of entire mRNA molecules by the construction of full-length-enriched cDNA libraries (Table 2). Ideally, almost all of the clones in the full-length-enriched cDNA libraries would contain entire coding sequences (CDSs). However, at the cloning step in the procedure of cDNA library construction, short transcripts that do not cover entire CDSs may be cloned preferentially to those containing functional CDSs. Degradation of RNA before library construction may also hamper the cloning of intact cDNA sequences in the libraries. Even though there are some incomplete transcripts in the ESTs, full-length-enriched cDNA libraries are much more valuable for determining the correct structures of functional mRNA than are cDNA libraries constructed by using normal methods, because the latter rarely yield intact full-length cDNAs [26].

**Table 2:** Correspondence to mammalian genes and estimated efficiencies of cloning of start codons of EST assemblies

	**Unique Gene ID (without HomoloGene ID)**	**Unique HomoloGene ID**	**Assemblies matched to protein sequences**			**Assemblies estimated to include start codons**		
				**Contigs**	**Singlets**		**Contigs**	**Singlets**
Human	13,691 (754)	12,911	64,011	12,056	51,955	47,229	9,635	37,594
Mouse	12,955 (730)	12,137	63,444	12,028	51,416	45,539	9,588	35,951
Cattle	13,445 (1935)	11,341	63,718	12,035	51,683	47,118	9,634	37,484
Dog	12,293 (763)	11,410	62,815	11,871	50,944	37,193	8,090	29,103
Pig	14,275		63,169	11,917	51,252	46,063	9,396	36,667

Numbers of genes that had unique NCBI Gene IDs and corresponded to contigs and singlets generated by assembly of expressed sequence tags (ESTs) are indicated. Also shown are the numbers that had unique Gene IDs in the NCBI HomoloGene database (a database of orthologs among species) and corresponded to the contigs and singlets generated. Numbers in parentheses indicate numbers of gene IDs that had no corresponding HomoloGene IDs. HomoloGene IDs in pigs are not indicated, because there is no HomoloGene ID database for pig genes.

EST assemblies were estimated to contain start codons if the length upstream of the matches (BLAST score >50) in the assemblies was greater than that between the start base of the coding sequence and the matched region of the corresponding gene. Numbers of assemblies (contigs and singlets) corresponding to protein sequences in humans, mice, cattle, dogs, and pigs are also shown.

Among the human genes matched to the assemblies, 12,937 corresponded to 12,911 unique NCBI HomoloGene IDs, which are indices of homologs among genomes of different eukaryote species [24]. This covered about two-thirds of all HomoloGene groups in humans (18,431 IDs; release 65). Furthermore, about 2000 additional genes were also included in the ESTs, as estimated from the numbers of genes without HomoloGene IDs (Table 2). In total, we estimated that more than 15,000 different genes were included in the ESTs thus obtained. However, the numbers of genes included in the EST assemblies might in fact increase because of gene duplication specifically occurring in the *Sus* genus.

The EST assemblies were mapped on the latest build of the draft sequence of the pig genome Sscrofa10.2 [6]. The entire summed length of chromosomes in the latest draft sequence of the pig genome (Sscrofa10.2) is 2596.6 Mb, and the 4562 scaffolds unplaced on any chromosome are a total of 211.9 Mb long [27]. Among the 17,183 contigs, 16,121 were mapped on pig chromosomes. Among the singlets, 86,464 of 120,928 were mapped on the chromosomes (Table 3). The assemblies were mapped to 39,445 different loci on pig autosomes and 1221 different loci on the pig sex chromosomes. In addition, 4463 loci corresponding to the assemblies were detected in scaffold sequences unplaced in any chromosomes (Figure 3A and Table 3). Mapping of loci corresponding to the EST assemblies on the draft sequence of the pig genome demonstrated that the ESTs were derived from regions throughout the whole chromosomes, and that the density of loci on the chromosomes showed periodic change within the chromosomes ( Additional file 1), possibly corresponding to the G- and R-bands of the chromosomes [28]. Although genes expressed ubiquitously in tissues (e.g., the gene encoding tubulin) were frequently observed, we were able to clone pig transcripts in the EST collection derived from more than 40,000 unique loci distributed throughout the whole genome. This shows that our EST resource is valuable for exploring pig gene sequences of interest in various areas of veterinary research.

**Table 3:** Mapping of pig EST assemblies on pig chromosomes

**Chromosome**	**Forward**			**Reverse**			**Total**		
	**Contigs**	**Singlets**	**Loci**	**Contigs**	**Singlets**	**Loci**	**Contigs**	**Singlets**	**Loci**
1	580	3343	1799	594	3346	1798	1174	6689	3597
2	639	2983	1452	646	2855	1365	1285	5838	2817
3	415	2248	1132	472	2574	1299	887	4822	2431
4	435	2049	1013	398	2112	1025	833	4161	2038
5	373	1774	870	375	2007	846	748	3781	1716
6	667	3188	1503	567	2826	1415	1234	6014	2918
7	526	2590	1211	631	3007	1039	1157	5597	2250
8	301	1637	851	215	1434	754	516	3071	1605
9	357	1966	1011	371	2082	1007	728	4048	2018
10	207	1118	555	200	1182	598	407	2300	1153
11	128	762	449	121	855	496	249	1617	945
12	388	2133	869	361	1759	896	749	3892	1765
13	489	2425	1293	515	2467	1321	1004	4892	2614
14	508	2693	1226	523	2716	1175	1031	5409	2401
15	284	1852	893	288	1828	797	572	3680	1690
16	147	817	498	139	894	509	286	1711	1007
17	348	1630	585	215	905	474	563	2535	1059
18	192	964	479	176	1127	479	368	2091	958
X	220	1018	591	216	1165	618	436	2183	1209
Y	4	14	6	1	6	6	5	20	12
Unplaced scaffolds	1077	7726	2251	812	4387	2212	1889	12,113	4463
Total	8285	44,930	20,537	7836	41,534	20,129	16,121	86,464	40,666

Numbers of EST (expressed sequence tag) assemblies and the corresponding independent loci mapped on the pig genome are shown for each chromosome. “Forward” and “Reverse” indicate that the assemblies were aligned on the chromosomes in the orientation from pter to qter or from qter to pter, respectively.

### Generation of the collection of pig cDNA clones, and complete sequencing of their inserts

The pig cDNA libraries used for the EST analysis were full-length–enriched libraries, which are ideal for determining the entire sequences of transcripts functioning as protein-encoding mRNA. In parallel with the EST analysis described above, we selected cDNA clones for the sequencing of entire inserts. The cDNA clones located at the forefront position in contigs generated by the assembly were selected for sequencing of the entire inserts, because we considered that they were the best candidates for clones carrying entire transcripts. As the EST analysis progressed, if cDNA clones located upstream of the clones already selected in the contigs appeared, we added these clones into the pipeline for sequencing of the entire inserts. On the other hand, among the singlets that did not join the contigs, there were many clones corresponding to human genes that had no counterparts among the clones selected from the contigs. We also selected these clones to ensure that the cDNA collection included a broad variety of genes. In total, we selected 42,047 clones as candidates for complete sequencing (31,545 clones from contigs and 10,502 clones from singlets).

Selected cDNA clones were sequenced by the primer-walking (PW) method [29]. cDNA clones that were difficult to sequence by PW were subjected to transposon-shotgun sequencing (TPS) with clone pooling [30-32]. To date, we have completed the sequencing of the entire inserts of 31,232 clones [DDBJ:AK230469–AK240615, AK343227–AK344223, AK344276–AK352511, and AK389169– AK399513, AK399520–AK401026]. We excluded the clones that had only repetitive sequences such as short or long interspersed nucleotide elements, and obtained 31,079 clones in a result (Table 1; 22,853 clones from contigs and 8226 clones from singlets), including 10,147 clones that have already been reported and presented in our cDNA database [19]. Among the candidate clones, 2169 were completely sequenced only by using universal sequencing primers (forward and reverse primers aligned to the vector sequence, and T_25_V primer). There were 25,629 cDNA clones sequenced by the PW method and 3500 by the TPS method (Table 4). The average length of the inserts of the cDNA clones thus sequenced was 1.63 kb. Sequencing by the TPS method effectively found longer inserts of the cDNA clones than by the PW method (Table 4), even considering the fact that one of the reasons why TPS was used on difficult-to-sequence clones was their length. The longest inserts of the clones sequenced by the PW and TPS methods were 4653 and 7293 bp, respectively. The TPS method is highly compatible with automation, because it does not require primer design; however, the total amount of sequencing work is much more than the PW method [30,31]. Therefore, application of TPS may be limited to clones with long inserts or for which many sequencing primers need to be designed.

**Table 4:** Distributions of lengths of cDNA clones completely sequenced by the primer walking and transposon shotgun sequencing methods

**Length (bp)**	**Sequenced using universal primersa**	**Primer walking**			**Transposon shotgun sequencing**
		**Once**	**Twice**	**Total**	
–1000	1174	1956	93	2049	156
1001–1500	928	9062	913	9975	519
1501–2000	67	6201	1843	8044	1058
2001–2500	0	2817	731	3548	766
2501–3000	0	919	428	1347	450
3000–3500	0	48	294	342	286
3501–4000	0	1	85	86	165
4001–	0	0	19	19	100
Total	2169	21,004	4406	25,410	3500
Average (bp)	907.6	1548.3	1959.6	1620.0	2163.3

Lengths of cDNA clones are shown according to the sequencing methods used. Clones sequenced by using the primer walking (PW) method are shown separately according to the number of primer walkings. Clones sequenced by using transposon shotgun sequencing (TPS) include those that were finished by the TPS method after being sequenced by the PW method.

^a^ Clones that were completely sequenced just by using universal primers (forward and reverse primers aligned to the vector sequence, and T_25_V primer).

### Genes corresponding to cDNA clones

BLAST similarity analysis of the inserts of the cDNA clones sequenced against the mRNA sequences of the NCBI RefSeq (release 49) revealed that the cDNA sequences corresponded to 11,298 human genes and more than 10,000 genes in each of mice, cattle, and dogs (Table 5). The functions of genes corresponding to more than 20,000 clones could be estimated by their similarity to human genes and classification according to Gene Ontology terms (Figure 3D). Among the human genes matched to the cDNA clones, 10,901 corresponded to 10,889 HomoloGene IDs (Table 5A); this accounted for 59% of all of the HomoloGene groups of human genes. Notably, more than 1000 genes corresponding to cDNA clones have not been classified in HomoloGene, and there may be duplication of gene loci originally occurring in the pig. Furthermore, it cannot be ruled out that the cDNA clones that showed no similarity to genes of other species encode genes that are functional in pigs. In total, we estimated that the cDNA thus sequenced represented more than 12,000 pig genes by comparative approach of mRNA sequences. In addition, cDNA clones corresponding to the same HomoloGene ID may be duplicated specifically on the pig genome. Although clarification of the correct number of genes included in our cDNA collection requires the completely sequenced genome sequence of the pig, we estimated the numbers of loci by using the currently publicized draft pig genome sequence (Sscrofa10.2).

**Table 5:** Mammalian genes corresponding to sequenced cDNA clones

	**Genes corresponding to sequenced cDNA clones**								**Sequenced cDNA clones containing full-length CDSs**					
	**Unique Gene ID (without HomoloGene ID)**		**Unique HomoloGene ID**		**Unmapped cDNA clones**				**cDNA clones(with corresponding loci on pig genome)**		**Unique Gene ID (with corresponding loci on pig genome)**		**Loci with Gene ID**	**Gene ID with cDNA clones unmapped on pig genome**^**a**^
					**Unique Gene ID (without Homolo Gene ID)**		**Unique HomoloGene ID**							
Human	11,298	(397)	10,889		604	(31)	572		12,498	(11,904)	5966	(5509)	5749	276
Mouse	10,688	(444)	10,197		553	(37)	513		12,354	(11,762)	5597	(5365)	5567	269
Cattle	10,881	(1329)	9487		568	(88)	479		12,622	(12,045)	5667	(5435)	5622	270
Dog	10,082	(469)	9556		522	(28)	492		9642	(9150)	4188	(4011)	4200	208
Pig	10,752				342				11,873	(11,420)	5244	(5116)	5343	182
Total^b^									14,616	(13,962)			6466	

Numbers of genes that had unique NCBI Gene IDs and corresponded to the sequences of the pig cDNA clones are shown at the left side of the table. Also shown are the numbers that had unique Gene IDs in the NCBI HomoloGene database (a database of orthologs among species) and corresponded to the sequences of pig cDNA clones. The numbers of unique Gene IDs corresponding to sequences of pig cDNA clones that were not mapped at any locations on the draft pig genome sequence (Sscrofa10.2) are also shown. In addition, the numbers of genes that had unique Gene IDs in the NCBI HomoloGene database and corresponded to the sequences of the unmapped pig cDNA clones are indicated. Numbers in parentheses indicate numbers of unique Gene IDs that had no corresponding HomoloGene IDs. Numbers of HomoloGene IDs in pigs are not indicated, because there is no HomoloGene ID database for pig genes. At the right side of the table, The numbers of cDNA clones estimated to contain full-length coding sequences (CDSs) of pig genes by alignment against the protein sequences of humans, mice, cattle, and dogs in the NCBI RefSeq database are shown in the first column. Pig protein sequences registered in RefSeq were used in this analysis. The numbers of unique NCBI Gene IDs corresponding to the cDNA clones and the numbers of loci corresponding to the cDNA clones examined using the draft sequence of the pig genome (Sscrofa10.2) are also indicated (“Loci with Gene ID”).

^a^ We counted the unique NCBI Gene IDs corresponding to cDNA clones that were not mapped on the draft sequence of the pig genome. If the Gene IDs corresponded to cDNA clones both mapped on, and not mapped on, the draft genome sequence, they were counted in both the “Loci with Gene ID” column and the “Gene ID with cDNA unmapped on pig genome” column.

^b^ Redundant cDNA clones corresponding to genes of more than one species were counted without repetition.

### Distribution of cDNA clones on the draft sequence of the pig genome

As mentioned above in the Background, the genome annotation process is greatly accelerated by the mapping of cDNA clones on the genomic sequence. We evaluated the usefulness of our collection of pig cDNA clones in genome annotation by mapping the clones onto the latest draft sequence of the pig genome (Sscrofa10.2). Among the 31,079 cDNA clones sequenced, 26,159 were mapped to 12,441 independent loci on the pig chromosomes. There were 12,042 loci mapped on the autosomes and 399 on the sex chromosomes (Figure 3B and Table 6). In addition, 3271 clones were mapped to 1453 loci on scaffolds that have not been localized on any chromosomes in Sscrofa10.2. Among the 1649 cDNA clones that were not mapped to pig chromosomes, and the unplaced scaffold sequences, 1114 clones corresponded to 604 unique human genes. Inclusion of those unmapped cDNA clones that corresponded to the genes of other mammals yielded a total of more than 600 unique genes corresponding to unmapped cDNA clones (Table 5). Taking these findings together, we estimated that our pig cDNA collection includes transcripts derived from about 15,000 different loci on the pig genome.

**Table 6:** Mapping of the pig cDNA clones sequenced in this study on pig chromosomes

**Chromosome**	**Forward**		**Reverse**		**Total**	
	**Clones**	**Loci**	**Clones**	**Loci**	**Clones**	**Loci**
1	1128 (164)	560 (134)	1197 (133)	594 (112)	2325 (297)	1154 (246)
2	1144 (138)	564 (123)	1180 (161)	563 (134)	2324 (299)	1127 (257)
3	744 (97)	380 (87)	871 (121)	468 (99)	1615 (218)	848 (186)
4	748 (88)	366 (71)	793 (96)	385 (81)	1541 (184)	751 (152)
5	710 (86)	310 (63)	685 (77)	313 (65)	1395 (163)	623 (128)
6	1184 (174)	552 (133)	1040 (130)	539 (115)	2224 (304)	1091 (248)
7	1013 (117)	437 (98)	1118 (125)	397 (97)	2131 (242)	834 (195)
8	563 (69)	267 (58)	393 (50)	209 (44)	956 (119)	476 (102)
9	699 (94)	339 (72)	673 (85)	339 (75)	1372 (179)	678 (147)
10	409 (48)	167 (37)	362 (37)	169 (32)	771 (85)	336 (69)
11	225 (26)	119 (25)	237 (22)	129 (20)	462 (48)	248 (45)
12	723 (89)	334 (76)	667 (68)	328 (60)	1390 (157)	662 (136)
13	922 (124)	431 (101)	890 (93)	467 (80)	1812 (217)	898 (181)
14	894 (101)	405 (80)	920 (116)	424 (90)	1814 (217)	829 (170)
15	568 (79)	281 (64)	518 (61)	247 (55)	1086 (140)	528 (119)
16	266 (35)	137 (26)	252 (39)	123 (35)	518 (74)	260 (61)
17	607 (70)	212 (59)	365 (47)	190 (38)	972 (117)	402 (97)
18	325 (32)	144 (29)	354 (38)	151 (30)	679 (70)	295 (59)
X	364 (56)	195 (49)	402 (73)	203 (55)	766 (129)	398 (104)
Y	5 (0)	2 (0)	1 (0)	1 (0)	6 (0)	3 (0)
Unplaced scaffolds	1854 (308)	747 (156)	1417 (162)	706 (135)	3271 (470)	1453 (291)
Total	15,095 (1995)	6949 (1541)	14,335 (1734)	6945 (1452)	29,430 (3729)	13,894 (2993)

Numbers of cDNA clones and the corresponding independent loci mapped on the pig genome are shown for each chromosome. “Forward” and “Reverse” indicate that the cDNA clones are aligned on the chromosomes in the orientation from pter to qter and from qter to pter, respectively. Numbers in parentheses show clones derived from pigs cloned from a female subjected to genome sequencing by the International Swine Genome Sequencing Consortium (Duroc 2–14).

Similar to the human genome, the pig genome is estimated to include 20,000 to 25,000 genes, because the genome size of pig is comparable to that of human. Our sequencing of the cDNA clones therefore covered slightly more than half of the entire gene set of the pig. The reason why thousands of genes were not included in our cDNA resources may be that the libraries were constructed with tissues of animals that were healthy and not subject to stressors such as infection or starvation. Genes that are highly expressed only during acute responses to pathogens or nutritional exhaustion might be difficult to clone in such libraries. In addition, if a gene is rarely expressed in a particular tissue (e.g., if its expression frequency among all the transcripts is less than 0.006%), then the probability that it will fail to be detected in the cloning of 10,000 transcripts will be more than 50%. To increase the number of cloned genes it would be necessary to normalize the libraries or use tissues derived from animals stimulated by particular stressors.

Genes encoded on the genome may be duplicated specifically in particular species but not in others. To detect duplication specifically occurring in the pig genome, we extracted the cDNA sequences with the longest open reading frames (ORFs) from among the clones that we sequenced here for the 12,441 putative loci on the autosomes and sex chromosomes in Sscrofa10.2. The extracted cDNA sequences were compared with human and cattle protein sequences deduced from the NCBI RefSeq (release 49). We estimated that 635 human protein sequences and 709 cattle protein sequences matched more than one putative locus on Sscrofa10.2. Conversely, the total numbers of loci estimated to be duplicated on Sscrofa10.2 in comparison with humans and cattle were 1358 and 1505, respectively. However, most of the potentially duplicated loci encoded shorter ORFs than their counterparts, implying that the loci were only pseudogenes or that they had arisen from the remaining sequencing errors in the genome sequence. Further refinement of the draft sequences of the pig genome will elucidate the duplication of genes occurring in the *Sus* genus.

The cDNAs thus analyzed were synthesized by reverse transcription using a poly-dT primer; therefore, most of the clones showed canonical mRNA features and had ORFs. Among the 13,894 loci mapped on the chromosomes and scaffolds, the representative (the longest) cDNA clones for 4144 loci showed no correspondence to the genes of humans, cattle, dogs, or mice. However, most of the cDNA clones with no obvious correspondence to the genes of other animals had apparent ORFs, and only 160 clones did not have ORFs for sequences more than 30 amino acids long. The average insert length of these 160 clones was 665 bp, whereas the average for all of the clones was much longer (1631 bp). These “non-coding” transcripts may be transcribed randomly and may have no function, although they may have a certain regulatory function on other protein-coding transcripts.

The draft sequence of the pig genome in its latest build (Sscrofa10.2) corresponds to the bacterial artificial chromosome (BAC) clones covering 98% of the physical map of the entire chromosome [5,6]. In our analysis, about 2% of the unique human genes corresponding to the cDNA sequences were not mapped to any chromosomes or unplaced scaffolds, showing that our estimate of the coverage of the whole genome by the draft sequence was correct. Refinement of the draft sequence of the pig genome will reveal the precise locations on the pig genome of the loci generating those transcripts that we cloned but that were not mapped, or that we mapped only on unplaced scaffolds.

The cDNA libraries that we used included seven libraries constructed by using animals that were cloned from a sow used for draft sequencing of pig genome (Duroc 2–14) [5-7]. A total of 4010 cDNA clones from four libraries were completely sequenced. Among them, 3729 were mapped to 2993 loci on the pig genome (Table 6B). These cDNA clones will be valuable for genome annotation of the draft sequence generated by the SGSC. In addition, comparison of the cDNA sequences of Duroc 2–14 clones with the draft sequences of the pig genome can be used to estimate the sequencing accuracy of the draft sequence and the frequency of RNA editing [33], although polymorphisms among the chromosomes of Duroc 2–14 hinder precise discrimination of such errors and edited bases. We roughly estimated such base changes by aligning cDNA sequences derived from Duroc 2–14 clones with the genome sequences. The region of each cDNA sequence that appeared most aligned was extracted, and the adenosine (A) to guanosine (G) base changes, which reflected the most representative A to I (inosine) RNA editing [34], were counted. To simplify the estimation, we investigated only A-to-G changes flanked by 5-base matches on both sides. Among the cDNAs of Duroc 2–14 clone pigs, 124 carried only A-to-G base changes, which totaled 142. In contrast, 91 cDNAs carried only G-to-A base changes, which totaled 97. Therefore, we estimated that about one-fourth of the inconsistency between G and A in the cDNAs and the draft sequence, respectively, is caused by RNA editing in the pig. Alignment of the 3′-UTR sequences of the cDNAs of Duroc 2–14 clone pigs showed differences of less than 0.4% from the draft genome sequence (data not shown). The differences thus detected included polymorphisms between different chromosomes and bases subjected to RNA editing. Furthermore, more than half of the aligned 3166 3′-UTRs (1883) were completely matched to the draft genome sequence. We therefore estimated that the actual error rate in the draft sequences of the pig genome was much less than 0.4%; the draft sequence was thus reliable.

### Coverage of coding sequences by cDNA clones on the pig genome

We expected that the collection of pig cDNA clones that we sequenced would include sequences covering the entire CDSs of pig genes. To estimate the numbers of cDNA clones covering entire CDSs, we investigated the coverage of those protein sequences of humans, mice, cattle, and dogs that showed the greatest similarity to the amino acid sequences deduced from the cDNAs. We also examined the distribution of the cDNA clones considered to cover entire CDSs on the pig chromosomes in the draft genome sequence (Sscrofa10.2). Among the cDNA clones sequenced completely, 14,616 were estimated to contain entire CDSs in their inserts. We estimated that these clones corresponded to 6466 different loci on the pig chromosomes (Table 5).

### Usefulness of the pig cDNA collection in genome annotation and other applications

The cDNA clones sequenced here were derived from libraries by methods that preferentially cloned intact RNA transcripts. About three-fourths of EST assemblies showing considerable similarity to known genes carrying the beginning of CDSs; we estimated that about half of the cDNA clones that were completely sequenced contained entire CDSs. An outline of the pig genome sequence is currently available, and use of the sequences of these expressed genes should help in precisely identifying the locations of genes on the genome and in determining the exon–intron structures of the genes. Along with the progress made in draft sequencing of the pig genome, automated annotation of the pig genome sequence has been conducted by the pipelines in Pre-Ensembl/Ensembl and publicized through the Pre-Ensembl/Ensembl database [35,36]. In the automated pipelines, about 30,000 pig cDNA clones were utilized [37]; most of these were derived from our pig cDNA sequencing project. Our data resources on pig-expressed genes have greatly contributed to prediction of the structures of genes on the draft sequence of the pig genome. In addition, the use of pig cDNA sequences that have been completely sequenced accelerates the process of manual refinement of automated genome annotation. Until now, many projects for full-length cDNA sequencing have been conducted in parallel with genome sequencing in eukaryotic species, and the results of these studies have contributed to our knowledge of gene locations and structures in target species such as humans and mice [9,38]. In fact, the pig cDNA sequences presented here have contributed greatly to the process of annotation of immune-related genes in the draft sequence of the pig genome by the Immune Response Annotation Group [10]. We expect that additional efforts to annotate other groups of pig genes will be accelerated by the use of our pig cDNA sequences.

One of the characteristics of the ESTs and cDNA sequences presented here is that the majority of the sequences were derived from intact mRNA with transcription start sites. This has great merit for exploring promoter sequences on the genome sequence [39]. The consensus sequences bound by transcription factors in the promoter sequences are generally well conserved among species; however, there are many variations in the binding-site sequences of transcription factors, and precise determination of the genomic region of the promoter sequence of each gene is essential for clarifying the efficiency of transcription in cells in response to stimuli [40]. Extraction of the upstream regions of the EST assemblies and cDNA sequences presented here, combined with direct evidence from, for example, ChIP-Seq studies [41], which will be accelerated by using the cDNA sequences for transcription factors in pigs, will enable the construction of a variable database for understanding transcriptional regulation of pig genes. Notably, we were able to completely sequence 1340 pig cDNA clones associated with “nucleic acid binding transcription factor activity,” as classified according to Gene Ontology (GO:0001071) (Figure 3D).

Another advantage of this collection of cDNA sequences is its usefulness for investigating alternative splicing events in pig genes. We mapped 29,430 cDNA clones to 13,894 different loci on the pig genome—that is, on average more than two different cDNA clones were derived from a single locus. Future studies should include a detailed exploration of splicing variants by using the cDNA sequences we have sequenced, together with the pig gene sequences presented by other groups.

The cDNA sequences and the ESTs themselves will also be useful in other studies, such as in gene expression analysis and in detecting polymorphisms in pig genes. A number of polymorphisms have been reported in mRNA sequences (particularly in CDSs), and it should be emphasized that there are many polymorphisms in CDSs that affect the functions of the molecules encoded by the genes that carry the polymorphisms. Our explorations of polymorphisms using the cDNA sequences and ESTs presented here have been useful in characterizing the genetic features of pig breeds and populations [18,42]. We have also investigated polymorphisms in genes encoding pattern-recognition receptors [43-46] and have demonstrated that some of the polymorphisms observed so far in commercial pig and wild boar populations truly affect the ligand-recognition ability of the molecules encoded by the genes [47-49]. In addition, many ongoing studies are revealing the potential associations of gene polymorphisms with economically important traits in pigs [50]. The use of pig gene sequences, including those presented here, will help greatly in promoting the exploration of polymorphisms that may be candidates for markers for selecting or breeding pigs with distinguished traits [1]. Furthermore, the gene sequences can be used directly to design probes for microarrays. We have developed oligomer microarrays by using sequences derived mainly from the ESTs and cDNA sequences presented here, and we have successfully elucidated the characteristics of changes in gene expression in pig subcutaneous preadipocytes [51]. Designing microarray probes with full-length cDNA sequences has benefits in terms of reliability, because the probes are highly specific to the target genes and there is clear evidence of correspondence between the probes and fully annotated genes. Full-length cDNA sequences will even be valuable in transcriptome analysis with NGS, which will become the mainstream method of expression analysis; these sequences will be useful in determining which short NGS reads belong to gene sequences that truly exert functions in organisms [16].

## Conclusions

Here, we demonstrated our attempts to collect pig-expressed genes by EST analysis and sequencing of entire cDNA clones using full-length-enriched cDNA libraries. We have so far accumulated 330,707 ESTs and 31,079 cDNA sequences. The ESTs and cDNA clones thus sequenced were respectively mapped to 40,666 and 13,894 different loci on the latest pig genome sequence Sscrofa10.2; they corresponded to more than 15,000 and 12,000 different genes of other species, respectively. The cDNA resource presented here is valuable for annotation of the draft sequence of the pig genome and for exploring promoter sequences on the genome. It will also be valuable for molecular biology–based analyses in pigs, for example for analyses of protein production *in vitro*.

## Methods

### Construction of cDNA libraries

Tissues for construction of the cDNA libraries were prepared from 10 crossbred [(Landrace × Large White) × Duroc] pigs, which are representative of those used for the Japanese pork market, and a Meishan animal, a breed representative of those used in China. The pigs were housed at the National Institute of Livestock and Grassland Science (Tsukuba, Ibaraki, Japan) [18,19]. We also used tissues from two Landrace and one Berkshire breed pig and one NIBS miniature pig [52]. In addition, we used a pig cloned from an animal of the Duroc breed (2–14) that was subjected to genome sequencing by the SGSC [5-7].

Using the collected tissues and cell populations, cDNA libraries were constructed by one of the following three methods. About two-thirds of the libraries (23) were constructed by using the oligo-capping method [21]. Fifteen of the oligo-capped cDNA libraries were constructed by using Gateway-compatible pCMVFL3 vector (Invitrogen, Carlsbad, CA, USA) [53], whereas the vector for the rest was pME18SFL3 (Toyobo, Osaka, Japan). Five cDNA libraries were constructed by using another method for constructing full-length cDNA libraries, namely the vector-capping method [20]. In total, 28 libraries were generated by methods using the 5′ cap structure, which is characteristic of intact mRNA. To compile the remaining four libraries, because only small amounts of RNA could be prepared from the tissues or cell populations, we used the SMART method and pDNR-LIB vector (Clontech, Palo Alto, CA, USA); this method selectively clones cDNAs that are synthesized as far as the 5′-end of the mRNA molecule [22]. All of the cDNAs were cloned into the vector unidirectionally. The library construction methods used for each tissue or cell population are shown in Table 1.

### EST analysis and clustering/assembling

The cDNA libraries thus constructed were subjected to EST analysis by single-pass sequencing from the 5Â´-ends of the respective clones. The EST reads obtained underwent basecalling using Phred; the vector sequences were screened by using the crossmatch program in the Phrap package [54,55]. Repetitive sequences and regions of low-complexity regions (e.g., poly(A) tracts) in the chromatograms thus generated were screened by using RepeatMasker [56] with RepBase [57] and in-house-generated Perl scripts. Clustering and assembly of sequences were performed with the TGICL package [58] with CAP3 [59]. Chromatograms that were not included in the contigs or that did not have regions containing more than 100 bases with Phred quality values ≥10 were discarded.

### Sequencing of entire inserts of cDNA clones

cDNA clones located at the most 5′ position in contigs generated by the assembling were selected for sequencing of the inserts. We also chose clones in singlets that did not join the contigs, provided that the clones corresponded to human genes with no counterparts among the clones selected from the contigs.

First, we sequenced the selected clones from the 5′-end with primer annealing with the vector sequence to confirm whether the correct clones had been selected. We also sequenced from the 3′-end with primer annealing with the vector sequence and T_25_V primer. The chromatograms that were obtained from the EST analysis and generated by sequencing from the 5′- and 3′-ends were subjected to basecalling with Phred and assembly by using Phrap. The contigs thus generated were screened with in-house Perl scripts to check for the low-quality regions (Phred quality values ≤ 25), and they were manually inspected for sequencing errors by using the Consed program [60]. Regions with low-quality or ambiguous bases were re-sequenced with primers (PW method; [29]) designed by using the Consed program with the “autofinish” option [60]. The procedure of inspection and sequencing of the remaining low-quality regions was performed twice or until no low-quality or ambiguous bases were observed. In addition to the PW method, we adopted another approach based on TPS, particularly for cDNA clones that could be difficult to sequence with the primer walking method [30-32,61]. With TPS, a large majority of the cDNA clones constructed by using the Gateway-compatible cloning vector (pCMVFL3; Toyobo, Osaka, Japan) were sequenced by using a combination of TPS and insert transfer with Gateway technology to reduce the number of shotgun clones with transposons in the vector sequence [31]. TPS was conducted with pooled DNA from two to 12 cDNA clones, as described previously [30,31].

### Computational analyses

The EST assemblies and cDNA clones were used in similarity analyses after interspersed repetitive sequences and low-complexity regions (such as polynucleotides and microsatellites) had been masked with the RepeatMasker program [56]. EST assemblies and cDNA clones on the pig genome sequence were mapped by using a BLAST similarity search [23] with the latest pig genome sequence, Sscrofa10.2 [6]. The best alignment of the respective query sequences, with a BLAST similarity score above 100 and identity above 90%, was anchored on the genome sequence. Overlapped alignments of cDNA sequences on the pig genome with opposite directions were regarded as different loci. The region of the locus was extended from the anchored alignment to both ends by using other alignments of the same query sequence meeting the following criteria: (1) the direction of the alignment was identical to that of the anchored alignments; and (2) the distance between the alignment from the region of the locus (which is a possible intron) was less than 1 Mb. The loci were regarded as identical if the locus regions after extension overlapped and were mapped in the same direction. Correspondence of the pig cDNA sequences to genes was investigated by BLAST similarity search using the mRNA or protein sequences in the NCBI RefSeq databases of humans, mice, cattle, dogs, and pigs [24]. The similarity was considered as positive if the BLAST score was more than 50. The presence of a full-length CDS in each pig cDNA sequence was estimated by BLAST similarity analysis, using those protein sequences showing the highest similarity in the NCBI RefSeq database. Two sequences were aligned without any filtering and masking. If the cDNA sequence was aligned with the specified protein sequence trimmed at both ends by fewer than 10 amino acids, then we considered that the cDNA contained a full-length CDS. EST assemblies and cDNA clones were classified according to the Gene Ontology terms by using the ontology file [62]. Classification according to Gene Ontology was conducted by using the similarity of cDNA clones to the mRNA sequences of human genes in the NCBI RefSeq and the correspondence between genes and Gene Ontology terms provided in NCBI Gene [24].

## Competing interests

The authors declare that they have no competing interests.

## Authors' contributions

HU designed the entire analysis and led the project. TM, DT, TEO, and HU conducted the EST analysis and sequencing of cDNA clones. LAR and LBS prepared the tissues of pigs cloned from the individual subjected to draft genome sequencing. Computational analysis was conducted by HU. HU wrote the draft, and all of the authors approved the manuscript.

## Supplementary Material

Additional file 1: **Density of genes on pig chromosomes, as demonstrated by localization of the EST assemblies.** Loci aligned by the expressed sequence tag assemblies within 5-Mb windows on the sequences of pig autosomes and the X chromosome (Sscrofa10.2) were counted. Each window slides by 100 kb. Solid lines indicate loci in the orientation from pter to qter on the chromosomes, and dotted lines indicate loci in the orientation from qter to pter. Click here for file
