# Supplementary material for: Large-scale sequencing based on full-length-enriched cDNA libraries in pigs: contribution to annotation of the pig genome draft sequence
Source: BMC Genomics. 2012 Nov 15;13:581. doi: 10.1186/1471-2164-13-581 (PMC3499286; doi:10.1186/1471-2164-13-581)
Supplement: Additional file 1: — Density of genes on pig chromosomes, as demonstrated by localization of the EST assemblies. Loci aligned by the expressed sequence tag assemblies within 5-Mb windows on the sequences of pig autosomes and the X chromosome (Sscrofa10.2) were counted. Each window slides by 100 kb. Solid lines indicate loci in the orientation from pter to qter on the chromosomes, and dotted lines indicate loci in the orientation from qter to pter. [file 1471-2164-13-581-S1.pdf]

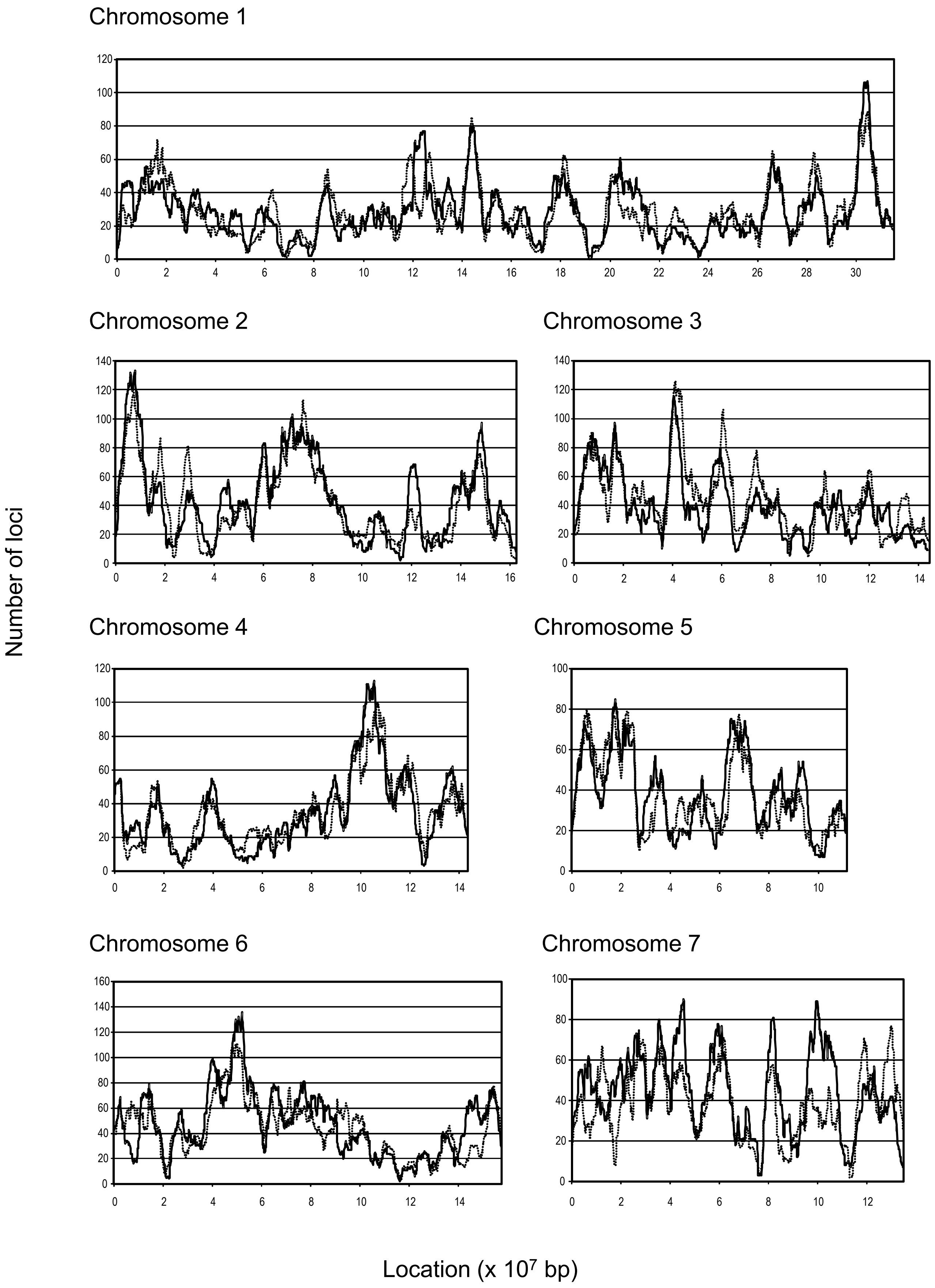

Additional file 1

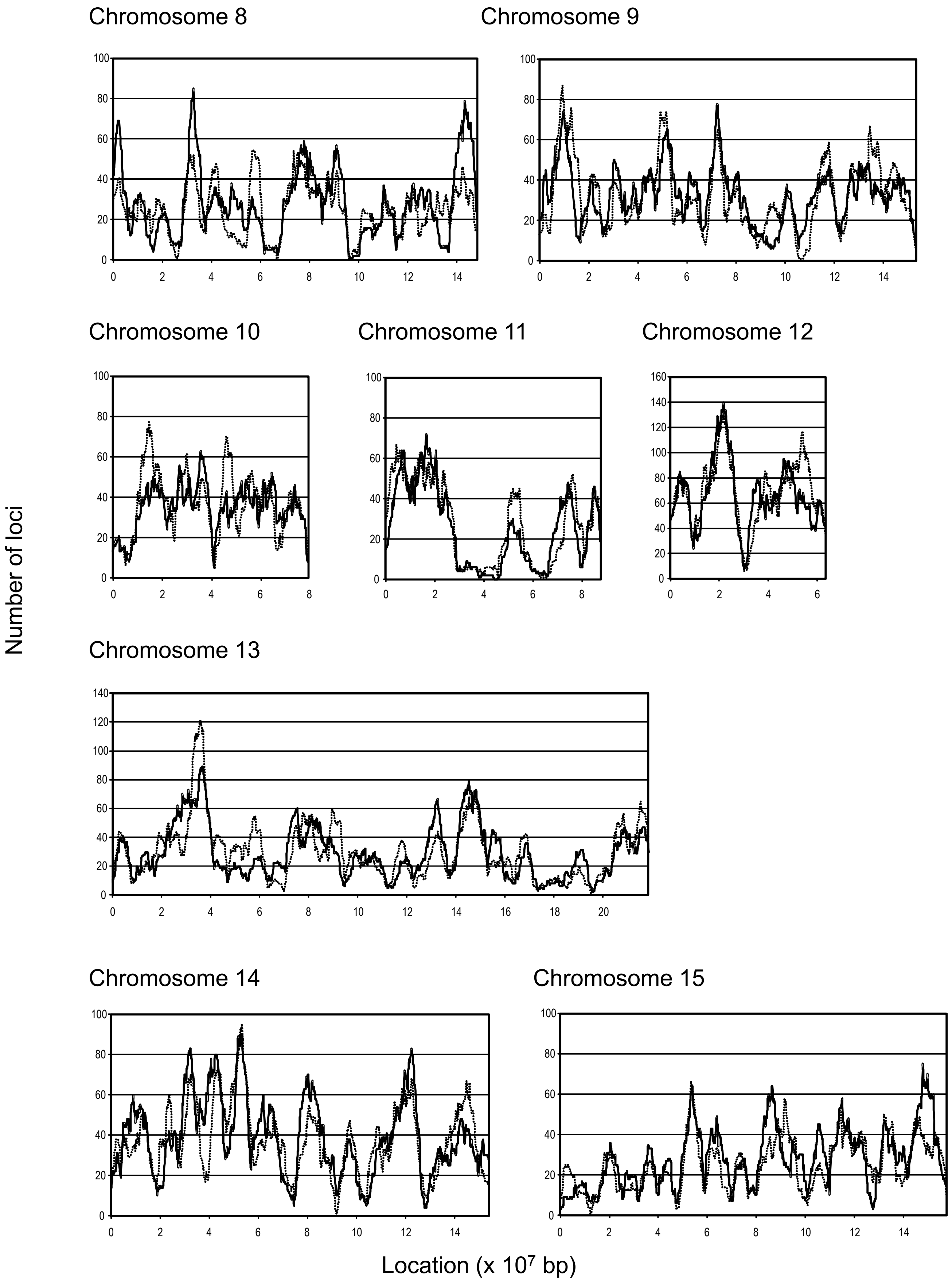

Additional file 1 (continued)

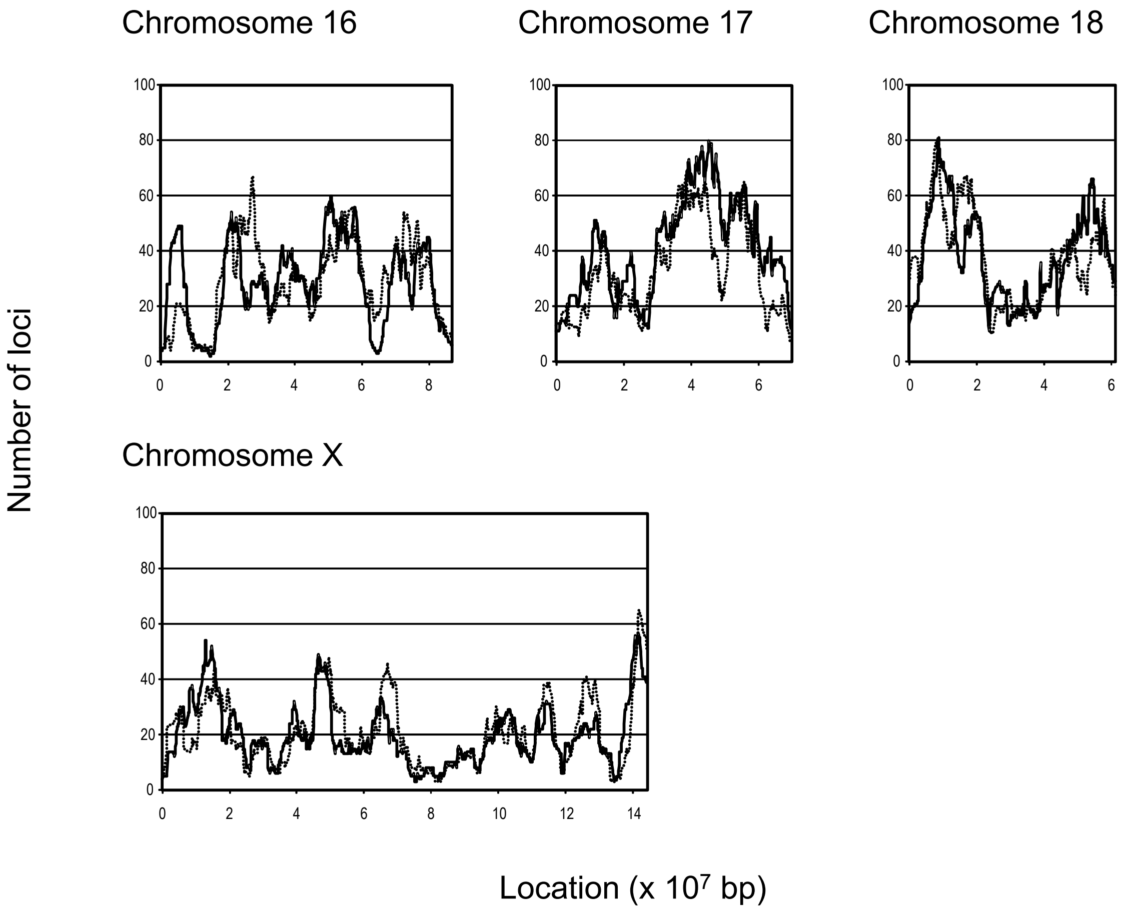

Additional file 1 - Density of genes on pig chromosomes, as demonstrated by localization of the EST assemblies.
Loci aligned by the expressed sequence tag assemblies within 5-Mb windows on the sequences of pig autosomes and the X chromosome (Sscrofa10.2) were counted. Each window slides by 100 kb. Solid lines indicate loci in the orientation from pter to qter on the chromosomes, and dotted lines indicate loci in the orientation from qter to pter.

Additional file 1 (continued)
